# Development of foot length in children with congenital clubfoot up to 7 years of age: a prospective follow-up study

**DOI:** 10.1186/s12891-021-04323-4

**Published:** 2021-05-27

**Authors:** Evgenia Manousaki, Anna-Clara Esbjörnsson, Gunnar Hägglund, Hanneke Andriesse

**Affiliations:** Department of Clinical Sciences, Lund University, Orthopedics, 221 85 Lund, Sweden

**Keywords:** Clubfoot, Foot length, Foot growth, Relapse

## Abstract

**Background:**

Clubfeet are typically shorter than normal feet. This study aimed first to describe the development of foot length in a consecutive series of children with congenital clubfoot and second to relate foot length to development of relapse and motion quality.

**Methods:**

Foot length was measured every 6 months in 72 consecutive children with congenital clubfoot (29 bilateral) aged from 2 to 7 years. The initial treatment was nonsurgical followed by standardized orthotic treatment. Foot length growth rate was calculated every half year. In children with unilateral clubfeet, the difference in foot length between the clubfoot and the contralateral foot was calculated. Motion quality was evaluated by the Clubfoot Assessment Protocol (CAP). Student’s t test, the Mann–Whitney *U* test and Spearman’s correlation were used for group comparisons. Bonferroni correction was used when multiple comparisons were performed.

**Results:**

Clubfeet were smaller *(P <* 0.001) than reference feet at all ages but had a similar growth rate up to age 7. Unilateral clubfeet with greater difference in size compared with the contralateral foot at the first measurement, relapsed more frequently (*P* = 0.016) and correlated with poorer motion quality (*r* = 0.4; *P =* 0.011).

**Conclusions:**

As previously reported, clubfeet were smaller than reference feet at all ages. The growth rate, however, was similar between clubfeet and reference feet. Children with unilateral clubfeet and greater foot length difference at 2 years of age had a higher tendency to relapse and poorer motion quality at 7 years of age, indicating that foot length could be used as a prognostic tool.

## Background

Congenital clubfoot includes hindfoot equinus and varus, midfoot cavus, and forefoot adduction [1, 2]. The characteristic abnormal foot positioning is a result of altered alignment, orientation and shape of bones and joints of the lower limb associated with soft-tissue abnormalities, such as muscle atrophy and a high proportion of adipose tissue [1, 2]. Even well-treated clubfeet are usually smaller due to bone hypoplasia and soft-tissue contractures [1–4].

In our clinical settings, we have observed irregular foot growth in relapsed clubfeet and poorer motion quality in children with smaller clubfeet compared with children with larger clubfeet. Foot size at birth can affect treatment results as it is sometimes more difficult to correct the initial deformity in smaller feet [4–7]. Hemo et al. found that severity score at baseline and number of casts needed for correction correlated with foot length before the start of treatment [7]. Foot size at end of growth can also depend on the treatment method [8–10]. Wallace et al. showed that in unilateral cases, nonsurgically treated clubfeet were 1.3 shoe sizes larger than surgically treated clubfeet, and that both surgically and nonsurgically treated clubfeet were significantly shorter than the contralateral feet at ages 10 to 12 years [8]. Kesemenli et al. showed that unilateral clubfeet were 9–15 mm shorter than the contralateral feet depending on the age and treatment method, with the surgically treated clubfeet showing a greater difference [10]. Additionally, Cooper and Dietz showed that unilateral clubfeet at maturity were, on average, 10 mm shorter than the contralateral feet [11]. While foot length measurements have been used during initial treatment and follow-up [12], to our knowledge there are no longitudinal studies evaluating clubfeet growth or the usability of such measurements.

The aims of this study were:

1. To describe the development of foot length from 2 to 7 years of age in children treated for congenital clubfoot.
2. To analyze the relationship between foot length and (a) relapse and (b) motion quality.

## Methods

Seventy-eight children were born with idiopathic congenital clubfoot within our catchment area from 1995 to 2007 and were invited to participate in this prospective longitudinal study. Children with eight examinations or more with at least 6 months between each visit were included. The median age at the first foot length measurement was 2 years (range, 2–3 years). The median number of measurements for each child was 10 (range, 8–11).

### Initial treatment and follow-up

**Fig. 1:**
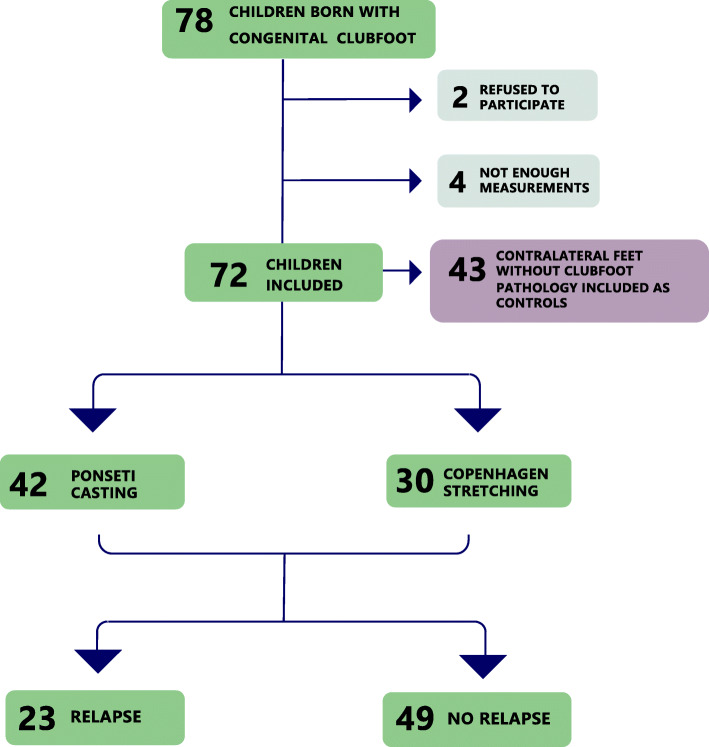
Flowchart of included children with clubfoot

**Fig. 2:**
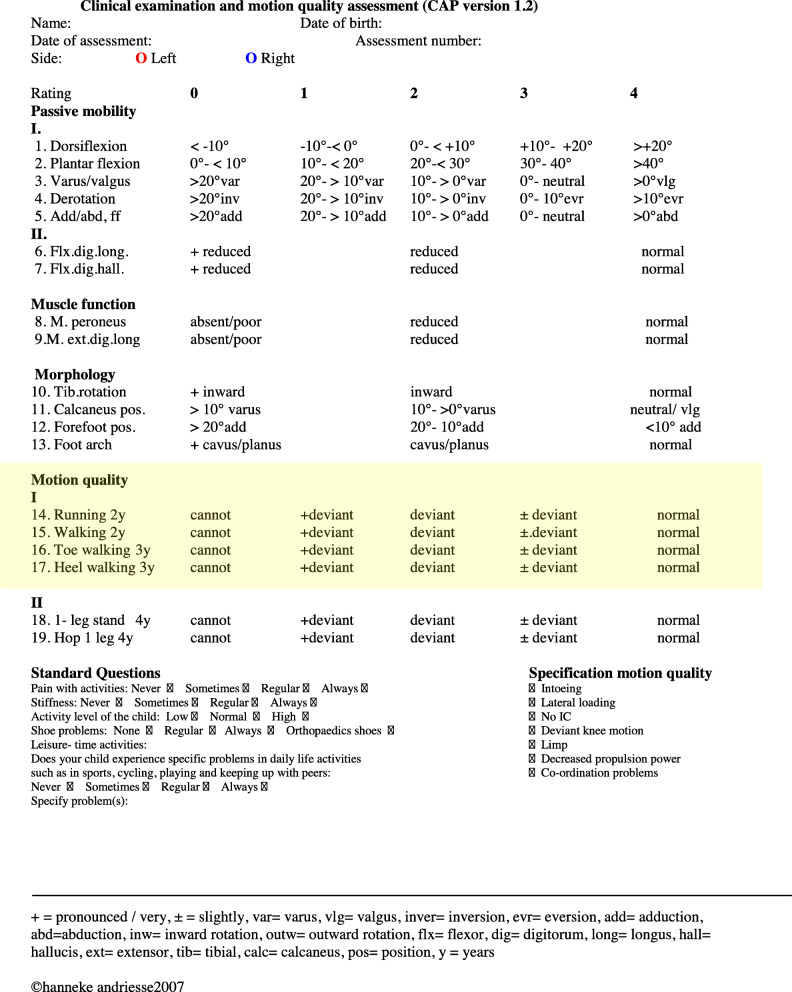
The Clubfoot Assessment Protocol. Motion Quality I highlighted. (With permission from Hanneke Andriesse) [15, 16]

Clubfeet were initially corrected using either the Copenhagen stretching method or the Ponseti casting technique (Fig. 1) [13, 14]. Percutaneous Achilles tenotomy was performed on 26 feet and posteromedial release on 22 feet. In both groups, all feet were fully corrected according the Clubfoot Assessment Protocol (CAP) (Fig. 2, domain ‘Mobility I’), before starting the orthotic treatment [15]. Until the age of 4, all children had the same orthotic treatment with individually made dynamic orthoses [17]. First, an individually made dynamic knee ankle foot orthosis (KAFO) was used. When the children’s independent gait was stabilized, a dynamic ankle foot orthosis (AFO) was applied and used nighttime [17]. These types of orthoses have shown good results, comparable with the results obtained with Foot Abduction Orthosis [17]. The children were followed according to a standardized protocol [16]. They were examined once every 1–3 weeks in the initial months, once every 3 months to the age of 2 years and then every 6 months from ages 2 to 7 years. The assessment included foot length measurement and physical examination according to the CAP (Fig. 2) [15, 18, 19].

### Foot measurements

**Fig. 3:**
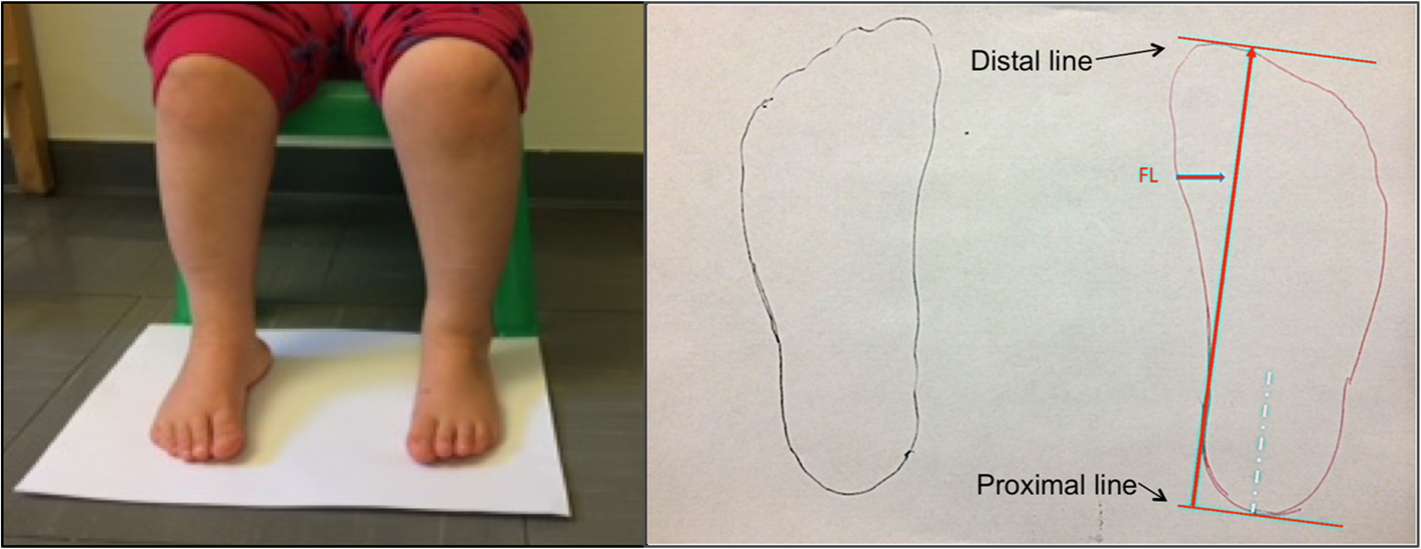
** a and b.** Foot length drawing and measurement. **a**: The child sits with the ankles, knees, and hips in 90° of flexion. A line is drawn around each foot with the pen kept vertical. **b**: Two parallel lines are drawn. The proximal line is drawn perpendicular to an imaginary line that passes through the middle of the hindfoot. The distal line is parallel to the proximal line. The distance between these lines is termed foot length

Foot length, including the contralateral nonaffected foot in children with unilateral clubfoot, was measured according to a standardized procedure. The child sat on a chair with ankles, knees, and hips in 90° flexion. A line was drawn around the foot with the pen kept vertical [20]. Parallel lines were drawn distally and proximally. To measure foot length from the drawings, the proximal line was drawn perpendicular to an imaginary line passing through the middle of the hindfoot. The distal line was drawn parallel to the proximal line, including the edge of the distal point of the foot. The distance between these lines was defined as foot length (Fig. 3). The same experienced physiotherapist (HA) made all the drawings. The first author (EM) later performed all foot length measurements.

The nonaffected foot in children with unilateral clubfoot served as the reference foot in the statistical analysis of foot length growth.

### Relapse

Relapse was defined as presence of one or more of the following: dorsiflexion < 0° with extended knees; subtalar joint mobility in valgus < 0°; foot outward rotation/abduction in relation to tibia < 5°; forefoot adduction > 10°; and/or in-toeing gait > 10°. Relapse was treated by reintroduction of orthosis, serial casting, surgery, or a combination of these treatments where appropriate.

### Motion quality

Motion quality was evaluated by the domain “Motion Quality I” (CAP_MQI_) from the CAP by a single assessor (HA). The CAP is a valid and reliable standardized multidimensional observer-administered test providing an overall profile of functional status for each side independently in children with clubfoot [15, 18, 19]. The CAP contains 19 items divided into subgroups (Fig. 2). The scoring for each item is graded from 0 (severe reduction/no capacity) to 4 (normal). Each grade is defined by specific criteria [16]. The CAP_MQI_ includes four items: walking, running, toe walking, and heel walking (0–16 points). The last CAP_MQI_ registered between the ages of 6 to 7 years was used in the analysis. Scores ≤ 12 were considered as poor clinical outcomes, according to previously established cutoff points [21].

### Data analysis and statistics

In children with bilateral clubfoot, data from either the right or left foot was included in the statistical analysis. The randomization was based on the child’s inclusion number, generating an equal number of left and right feet. Hence, only one clubfoot from each included child was used in the analysis.

Foot length growth percentage (FLG%) was calculated by subtracting the previous foot length value from the current value. That value was then divided by the previous foot length value and multiplied by 100 to express foot length growth in percent. For example, the FLG% between ages 2 and 2.5 years equals (foot length at 2.5 years–foot length at 2 years) / foot length at 2 years ⋅ 100.

Foot length difference percentage (uniFLD%) was calculated for every child with a unilateral clubfoot by subtracting the foot length of the clubfoot from the contralateral nonaffected foot. The difference was divided by the contralateral nonaffected foot length value and multiplied by 100 to express foot length difference in percentage.

Statistical analysis was performed using IBM SPSS Statistics software (version 25; IBM SPSS, Armonk, NY, USA). Student’s t test was used to analyze the differences in foot length and FLG% between clubfeet (unilateral and bilateral-one randomly selected foot from each child included) and reference feet and between clubfeet with and without relapse. In unilateral feet the paired Mann–Whitney Wilcoxon test was used to compare the distribution of uniFLD% at initial measurement between children with and without relapse before the age of 7. Spearman correlation test was used to analyze the correlation between uniFLD% at initial measurement and CAP_MQI_ at the age of 7. A *P* value of below 0.05 was considered statistically significant. For multiple comparisons, the Bonferroni correction was used. The α was set at 0.05 and the *P* value was adjusted to 0.005. Correlations were interpreted according to Cohen’s method as low (0 to ± 0.29), moderate (±0.30 to ±0.49), and strong (±0.5 to ±1.0) [22].

Written informed consent was obtained from the legal guardians of all participants and the study was approved by the local ethics committee (LU-667-03).

## Results

Seventy-two children were included in this study (55 boys and 17 girls, 43 unilateral and 29 bilateral). Thirty children were initially treated with Copenhagen stretching and 42 with Ponseti casting. The median Dimeglio score was 10 (range 7–14). There were no statistically significant differences in relapse rate, baseline severity, gender, or laterality between the treatment groups. Children treated with the Copenhagen stretching method underwent significantly more posteromedial releases (17/30, 60 %) at the end of the initial treatment than the children treated with Ponseti casting (5/42, 12 %) (*P* < 0.001).

### Development of foot length and foot growth

Clubfeet were smaller than reference feet at all ages (*P* < 0.005). Clubfeet grew from a mean and standard deviation (SD) of 134 ± 7 mm at the age of 2 years to a mean of 183 ± 12 mm at 7 years, whereas reference feet grew from a mean of 140 ± 8 mm to a mean of 193 ± 12 mm over the same period (Table 1). FLG% decreased from 5.4 % to 2 years of age to 2.3 % at 7 years of age (Table 2). Foot length and FLG% were similar among children treated with Copenhagen stretching and Ponseti casting. There were no statistically significant differences in baseline severity, gender, foot length or growth between children with unilateral and bilateral clubfeet.

**Table 1:** Foot length (mm) in reference feet* and in clubfeet** at different ages

*Age (years)*	*Reference*		*Clubfeet*		*P value*
	***n***	***mean (SD)***	***n***	***mean (SD)***	
**2**	36	140 (8)	61	134 (7)	*0.001*
**2.5**	38	148 (8)	67	141 (8)	*0.000*
**3**	41	155 (9)	70	147 (9)	*0.000*
**3.5**	37	160 (9)	62	152 (10)	*0.000*
**4**	43	165 (9)	71	157 (9)	*0.000*
**4.5**	42	171 (9)	70	161 (10)	*0.000*
**5**	40	176 (10)	69	165 (10)	*0.000*
**5.5**	33	179 (10)	57	169 (10)	*0.000*
**6**	37	185 (9)	64	174 (10)	*0.000*
**6.5**	37	189 (9)	59	179 (9)	*0.000*
**7**	35	193 (11)	60	183 (12)	*0.000*
n, number of measurements; SD, standard deviation. P values after Student’s t test. Significance set up to P < 0.005 after Bonferroni correction. *Contralateral feet in children with unilateral clubfoot. ** unilateral and bilateral (*one randomly selected foot from each child was included)*

**Table 2:** Foot length growth percentage in reference feet* and in clubfeet**

*Age interval (years)*	*Reference*		*Clubfeet*		*P value*
	***n***	***mean (SD)***	***n***	***mean (SD)***	
***2–2.5***	36	5.4 (4)	61	5.4 (4)	*0.971*
***2.5–3***	36	5.0 (3)	65	4.4 (3)	*0.300*
***3–3.5***	37	3.5 (2)	61	3.9 (2)	*0.362*
***3.5–4***	38	3.3 (2)	62	2.9 (2)	*0.329*
***4–4.5***	42	3.4 (2)	69	2.8 (2)	*0.077*
***4.5–5***	39	2.9 (2)	67	3.0 (2)	*0.900*
***5–5.5***	32	2.8 (2)	55	2.5 (2)	*0.419*
***5.5–6***	30	2.4 (2)	52	2.7 (2)	*0.464*
***6–6.5***	34	2.4 (2)	55	2.9 (2)	*0.203*
***6.5–7***	31	2.6 (2)	49	2.3 (2)	*0.688*
***Total (2–7)***	*28*	*40 (6)*	*49*	*38 (7)*	*0.216*
n, number of measurements; SD, standard deviation. P values after Student’s t test. Significance set up to P < 0.005 after Bonferroni correction. *Contralateral feet in children with unilateral clubfoot. ** unilateral and bilateral (*one randomly selected foot from each child was included)*					

In children with unilateral clubfoot, the foot length difference percentage (uniFLD%) between the clubfoot and the contralateral nonaffected foot was approximately 3 % at 2–2.5 years of age, and 5 % from 2.5 to 7 years of age.

### Foot length in relation to relapse

**Fig. 4:**
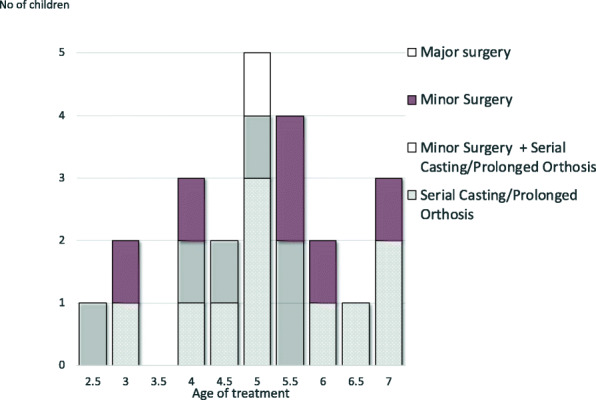
Treatment methods for relapse related to age

**Fig. 5:**
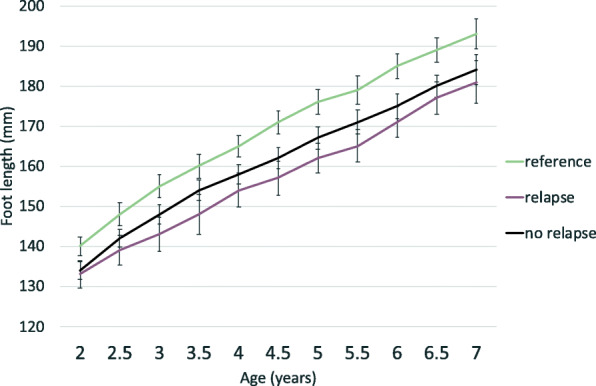
Foot length development in reference feet (green), clubfeet with relapse (pink), and clubfeet without relapse (dark blue). The gray lines show the confidence interval

Before the age of 7 years (median 5 years), 23 of the 72 children (32 %) were treated for relapse. Seventeen children had one relapse and six children had two relapses (Fig. 4). No differences in relapse rate were seen with respect to initial treatments, unilateral or bilateral involvement or gender. No statistically significant differences were found in foot length or in FLG% between children with and without relapse (Table 3; Fig. 5).

**Table 3:** Foot length development (mm) in children with clubfeet* with and without relapse

*Age (years)*	*Relapse*		*No relapse*		*P value*
	***n***	***mean (SD)***	***n***	***mean (SD)***	
***2***	17	133 (7)	44	134 (8)	*0.519*
***2.5***	21	139 (9)	46	142 (8)	*0.254*
***3***	22	143 (10)	48	148 (8)	*0.051*
***3.5***	18	148 (11)	44	154 (8)	*0.033*
***4***	22	154 (10)	49	158 (9)	*0.065*
***4.5***	23	157 (10)	47	162 (9)	*0.032*
***5***	23	162 (9)	46	167 (9)	*0.024*
***5.5***	20	165 (9)	37	171 (9)	*0.05*
***6***	21	171 (9)	43	175 (10)	*0.074*
***6.5***	20	177 (9)	39	180 (9)	*0.314*
***7***	20	181(12)	40	184 (12)	*0.354*
n, number of measurements; SD, standard deviation, P values after Student’s t test. Significance set up to P < 0.005 after Bonferroni correction. * unilateral and bilateral (*one randomly selected foot from each child was included)*					

Children with unilateral clubfoot that relapsed before 7 years of age had larger uniFLD% at their first measurement around 2 years of age (5 %; interquartile range (IQR), 3–7 %), compared with those without relapse (3 %; IQR, 0.75–5 %) (*P* = 0.016).

### Foot length difference in relation to motion quality in children with unilateral clubfoot

The median CAP_MQI_ at the last measurement of all clubfeet (median age, 7 years; range, 5.5–7 years) was 13 (IQR, 12–15), with 76 % of the children having scores above cutoff, indicating good motion quality [21]. In children with unilateral clubfoot, a moderate correlation was found between the uniFLD% at baseline and CAP_MQI_ at 7 years of age (*r* = 0.4; *P* = 0.011*).*

## Discussion

This prospective longitudinal study included a consecutive cohort and aimed to evaluate the development of foot length and foot growth in children with clubfoot from 2 to 7 years of age. The study cohort is representative with respect to unilateral and bilateral involvement, gender distribution, and relapse rate [23–28]. We found that clubfeet were shorter than reference feet at all ages. However, clubfeet growth after the age of 2 years was similar to the growth of reference feet. Children with unilateral clubfeet, with a greater difference in foot length at initial measurement, relapsed more frequently and had poorer motion quality at 7 years of age.

Muller et al. analyzed foot length development in 10,000 typically developed children and found an average increase in foot length of 51 mm between ages 2 and 7 years [29]. These results are consistent with our findings where reference feet grew at an average of 53 mm between ages 2 and 7 years and clubfeet grew an average of 49 mm. Even though clubfeet were smaller compared with reference feet, the percentage foot length growth of reference feet and clubfeet was similar between 2 and 7 years of age. This could partly be explained by decreased bone hypoplasia as suggested by Beck et al. [3]. They found that bone hypoplasia decreased with age when evaluating children with clubfeet between the ages 2 to 4. In our study, a minor growth slowdown was observed in all clubfeet at ages 3.5 to 4.5 years, possibly caused by the absence of daily stretching when the orthosis treatment ended (Table 2).

The relapse rate in this study was 32 %. Previous studies have reported relapse rates ranging from 3.7 % up to 53 % depending on the initial treatment method, bracing protocol, follow-up time, and relapse criteria [23–27]. Most (87 %) of the relapses in this study were observed after completion of orthosis treatment. This coincides with increased variation in foot length and foot growth, indicating the importance of daily stretching to prevent relapse. Thus, careful follow-up of clubfoot development after ending orthosis treatment is imperative to detect early relapse.

Small clubfeet have been associated with difficulties at initial correction and increased risk of relapse [4, 6, 7]. We found that a greater difference in foot length at baseline in children with unilateral clubfeet was related to an increased number of relapses and worse motion quality score at the age of 7. Our findings, based on children treated with the same follow up-protocol, indicate that foot length at the age of 2 years could be used as a prognostic tool. For example, the foot size at the age of 2 could be considered when the decision to continue or dismiss the orthotic treatment at the age of 4 is taken. Estimating the exact relationships between foot length at an early age and risk of relapse and poor motion quality later in life could be of value for clinical treatment and follow-up planning. In addition to the predictive value of systematically measuring foot length, foot drawings are an easy and inexpensive method to monitor foot growth and shape, providing informative visual feedback on clubfoot development. Furthermore, it is easily understood by both patients and parents.

As multiple comparisons were made, the Bonferroni correction was applied. Without this correction, the differences in length and growth between clubfeet with and without relapse were significant at around the ages of 3 to 5 years, when most of the relapses occurred (Table 3). This finding is consistent with our clinical observation that clubfeet growth occasionally slows down during relapse and normalizes after appropriate intervention. However, we cannot exclude the possibility that a type 2 error occurred when foot length and FLG% were compared [30].

In this study, the contralateral feet were used as reference feet, which could be considered a limitation [31]. However, the reference feet in our study did not differ in size from typically developing feet as described in the literature [29]. The intra- and interrater reliability have not yet been established for measurements made using the foot drawing method. In our study, the same assessors performed all drawing (HA) and length measurements (EM) to minimize operator error.

Another limitation is that the treatment methods used in our cohort are not gold standard and the results need to be confirmed in children treated within the strict Ponseti protocol. On the other hand, clubfeet in our study showed the same growth rate as reference feet, and similar relapse rate as clubfeet treated with strict Ponseti protocol, indicating generalizable results.

## Conclusions

As previously reported, clubfeet were smaller than reference feet at all ages. The growth rate, however, was similar between clubfeet and reference feet. Children with unilateral clubfeet and greater foot length difference at 2 years of age had a higher tendency to relapse and poorer motion quality at 7 years of age, indicating that foot length could be used as a prognostic tool.

## Data Availability

The datasets used and analyzed during the current study are available from the corresponding author on request.

## Abbreviations

CAP: Clubfoot Assessment Protocol
CAP_MQI_: Clubfoot Assessment Protocol Motion Quality I
FLG%: Foot length growth percentage
uniFLD%: Unilateral foot length difference percentage
SD: Standard deviation
